# An investigation of feed-forward and feedback eye movement training in immersive virtual reality

**DOI:** 10.16910/jemr.15.3.7

**Published:** 2023-06-12

**Authors:** David J. Harris, Mark R. Wilson, Martin I. Jones, Toby de Burgh, Daisy Mundy, Tom Arthur, Mayowa Olonilua, Samuel J. Vine

**Affiliations:** School of Public Health and Sport Sciences, University of Exeter, UK; Defence Science and Technology Laboratory, Salisbury, UK; Cineon Training, Exeter, UK; RINA, Swindon, Wiltshire, UK

**Keywords:** Eye movement, eye tracking, VR, skill acquisition, military, defence

## Abstract

The control of eye gaze is critical to the execution of many skills. The observation that task experts in many domains exhibit more efficient control of eye gaze than novices has led to the development of gaze training interventions that teach these behaviours. We aimed to extend this literature by i) examining the relative benefits of feed-forward (observing an expert’s eye movements) versus feed-back (observing your own eye movements) training, and ii) automating this training within virtual reality. Serving personnel from the British Army and Royal Navy were randomised to either feed-forward or feed-back training within a virtual reality simulation of a room search and clearance task. Eye movement metrics – including visual search, saccade direction, and entropy – were recorded to quantify the efficiency of visual search behaviours. Feed-forward and feed-back eye movement training produced distinct learning benefits, but both accelerated the development of efficient gaze behaviours. However, we found no evidence that these more efficient search behaviours transferred to better decision making in the room clearance task. Our results suggest integrating eye movement training principles within virtual reality training simulations may be effective, but further work is needed to understand the learning mechanisms.

## Introduction

To behave adaptively and execute complex behaviours in dynamically changing environments, an organism must selectively sample from the wealth of information available from the sensory array. During complex decision-making scenarios, such as team sports or military combat, vision is particularly important for providing information to support continuous sequences of sensorimotor operations that satisfy current behavioural goals (11; 22; 45). Consequently, learning to optimally control the gaze system is critical to performance in these situations (10; 25; 30). Studies of gaze selection in natural environments point to a consistent set of principles underlying eye guidance, involving (1) behavioural relevance (based on reward mechanisms), (2) uncertainty about the state of the environment, and (3) learned prior models of the self and surrounding context (23; 45; 51). With experience, and through training, task experts learn to strategically direct their gaze to maximise information acquisition (6). A large body of literature has illustrated that it is possible to accelerate this learning of gaze control and dependant motor skills using *eye movement training* (15; 27; 39; 43; 53). The current study developed and tested a novel method of implementing eye movement training in immersive virtual reality (VR) to further our understanding of gaze training methodologies.

In this work, we built upon previous research that has trained perceptual cognitive skills – the ability to identify and environmental information, integrate it with existing knowledge and execute appropriate actions (38) – using a method known as *feed-forward eye movement training* (26, 27; 33; 39; 42; 43; 53). Feed-forward eye movement training (FFEMT), also known as *Eye Movement Modelling Examples* (EMME) in some contexts, aims to teach the gaze strategies of expert performers to more novice trainees to accelerate their learning. This may be achieved through explicit instruction about where to direct vision or, more commonly, through the use of point-of-view eye tracking videos (where an overlaid gaze cursor indicates where task experts direct their fixations and scan paths; 39; 42). FFEMT is appropriate for skills that have an underlying visual guidance component and has been applied to tasks like laparoscopic surgery (58) and scanning medical images for defects (14). Much of the work in this area has originated from sport where FFEMT has been applied to aiming skills like golf putting and basketball free throws (8; 17; 56). In these types of skills, fixations are tightly coupled, temporally and spatially, to well-learned motor actions (31) and FFEMT has been very successful in accelerating learning and making performers more robust to performance pressure (54, 53; 56). FFEMT has also been successfully applied to military skills. For instance, in a simulated maritime marksmanship task with a decommissioned general-purpose machine gun, Moore et al. (42) found that participants given FFEMT showed superior gaze control and shooting performance than those given technical instruction.

A novel aspect of the current work was the integration of eye movement training with immersive virtual reality. VR, and related technologies (e.g., augmented reality and mixed reality), are becoming common methods of training in physical rehabilitation (1), military (34), nuclear safety (16), and sporting (18) settings. VR is becoming more accessible due to the rapid development of commercial head-mounted displays (HMDs) and offers potential training benefits, such as improved safety, reduced cost, and greater access to training when physical spaces are a limited resource. In addition to these more practical advantages, there may be pedagogical benefits arising from the possibility of automated performance monitoring and feedback capabilities. As commercially available HMDs now often include built-in high resolution eye tracking for the purposes of optimising the visual display, they afford an opportunity for seamless implementation of eye movement training principles in VR training applications (18).

In the present work we aimed to test whether FFEMT in VR could be effective in the context of military room clearance, a complex visuomotor and decision-making skill. During room clearance drills (also known as close-quarter battle), operators are required to enter a room, scan the area for threats, identify threatening and non-threatening targets, determine appropriate use of force, and accurately aim a weapon. Consequently, efficient use of vision is important for quickly extracting information from the room. In a more passive virtual room searching task (no ‘use of force’ decision making), Harris et al. (19) found that visual search skills could be trained in VR through trial and error learning, and that faster room searches were characterised by more efficient visual search patterns (e.g., lower search rates and reduced scanning entropy). Here, we aimed to extend this work by explicitly teaching search patterns using FFEMT.

Additionally, we aimed to extend previous eye movement training work by adopting a method of *feed-back eye movement training (*FBEMT). Instead of showing trainees the eye movements of an expert before they complete a task, FBEMT replays trainees their own eye movements that were performed during the task to enable them to learn from their mistakes (54). The mechanisms underpinning FFEMT are thought to involve a mostly implicit development of efficient visual guidance by adopting the search strategy of the expert (52), which cues the trainees attention towards important areas of the visual scene (12; 26). By contrast, the mechanisms behind FBEMT are less clear. Previous work on observational learning has shown that allowing trainees to observe their own mistakes supports the development of error detection and correction mechanisms (5; 7), which could be the mechanism for learning from FBEMT. Consequently, FBEMT may provide different but complementary support to learning, yet it has received limited attention in the literature (although see use as a debriefing/feedback tool in emergency resuscitation: 50, 49). Using a population of military recruits from the British Army and Navy, we compared both FFEMT and FBEMT to training as usual to determine whether these methods could accelerate training.

### Hypotheses

Based on previous research into FFEMT and FBEMT (26; 32; 54, 53), it was predicted that participants who were given VR eye movement training would show more efficient visual search behaviours at post-test than a control group, and greater improvements in simulated task performance. It was also predicted that FFEMT and FBEMT could lead to improved performance in the virtual room clearance task (fewer civilians shot, fewer targets missed, faster clearances).

## Methods

### Participant and public involvement and engagement

While this project sought to examine widely applicable training principles, it was targeted at a specific skill and population use-case. Therefore, we adopted participant and public involvement and engagement practices to guide the work and ensure that those personnel with an interest in improved training contributed to how the research was designed, conducted, and disseminated. We held a series of stakeholder workshops where the plans for the virtual environment and the experimental trial were presented to subject matter experts from across UK Defence and Security agencies (Policing, Army, Navy, and Air Force). These individuals therefore had experience of training design and research into training design, in a defence and security context, but were not necessarily experienced in the use of VR technologies for training. During these workshops, it was identified that the proposed training methods should be aligned with existing ‘real-world’ training principles, so that we could contextualise any performance adaptations in a valid and meaningful way. Though a number of potentially relevant skills were initially identified by the stakeholders, room clearance was selected as the most widely applicable across all forces and was deemed to be suitable for eye movement training (given previous research findings, e.g. 42). Subject matter experts also had significant input into the design of the virtual environment, which was developed to closely replicate many existing training facilities.

### Design

**Figure 1.**
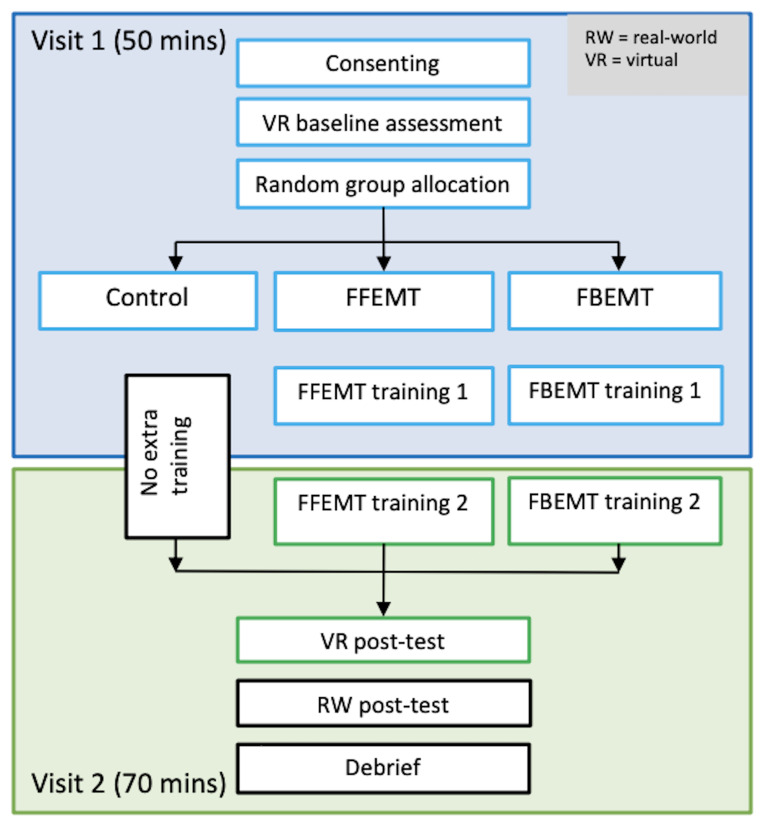
Trial design overview. The two visits took place on consecutive days.

The study adopted an independent groups design (see design overview in Figure 1). Participants were randomly allocated to one of the three training groups (FFEMT, FBEMT, or control) and performed pre- and post-tests in the virtual environment. Following the recommendations of Karlsson and Bergmark (28) for selecting the relevant causal contrast to the treatment group in randomised controlled trials, control participants were assigned to training as normal. Training as normal consisted of continuing with their close quarter battle training using synthetic physical environments, but no additional FBEMT or FFEMT. As the purpose of this trial was to determine whether FFEMT or FBEMT could be an effective addition to current training, training as usual provided the relevant baseline.

Participants were also asked to complete a real-world room clearance task, but due to practical difficulties of running these in military locations with suitably qualified training personnel, the procedures could only be completed on a sub-group of the overall sample. Given that this element of the study was likely underpowered and unable to provide clear conclusions, we have reported it only in the supplementary materials (see https://osf.io/qn2g4/).

### Participants

Participants were recruited from two military groups: British Army personnel from the Infantry Battle School (IBS; Powys, Wales) and Royal Marine Commandos from the Commando Training Centre Royal Marines (CTCRM) at Lympstone (Exmouth, Devon). Both groups undertake close quarter battle training and learn similar techniques for entering and clearing a room. As the Royal Marines were recruited from an earlier stage of training, population group was initially included as a covariate in all analyses (detailed below), however this was only retained if it displayed a significant relationship with the dependent variable. Although a recruitment target of 51 participants was initially set (see supplementary materials for power calculation: https://osf.io/qn2g4/), this was an opportunity sample and a total of forty participants were able to be enrolled in the study (26 Royal Marines and 14 British Army; see Table 1). Two participants (one in the FFEMT group and one in the control group) did not return for the second testing visit, which left 38 complete data sets for pre- and post-tests in the VR task (see Table 1). Therefore, a sensitivity analysis was performed to establish the kind of effect sizes we were able to detect (Lakens, 2021). This analysis indicated that the 38 data sets were sufficient to detect effects up to ηp2 = 0.28 with 90% power, η_p_^2^ = 0.21 with 75% power, and ηp2 = 0.16 with 60% power, in a 3 (group) x 2 (time) ANOVA. All participants gave informed consent prior to taking part and the design was reviewed by both the Ministry of Defence Research Ethics Committee and a University research ethics panel.

**Table 1.** Summary of the Number of Participants from Each Population Assigned to Each Experimental Group.

	Mean age (SD)	FFEMT	FBEMT	Control	Total
Army	28.1 (6.1)	5	4	5	14
Royal Marines	23.0 (3.0)	8	9	9	26
	**Total**	13*	**13**	14*	**40**

*1 participant in the FFEMT group and 1 in the Control group did not return for a second visit and therefore these are not included in the analyses

### Materials

#### VR equipment

**Figure 2.**
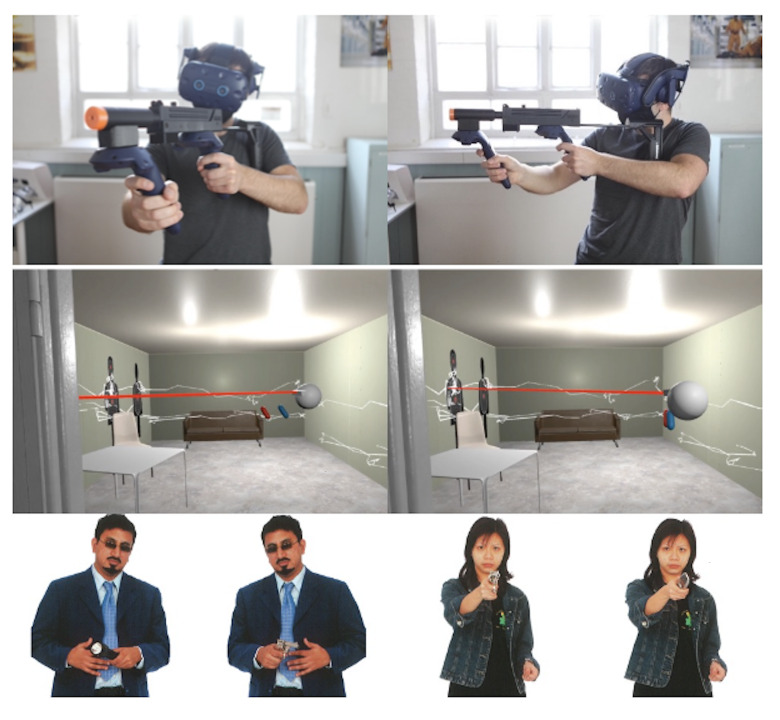
VR Hardware and Software. Top: HTC Vive Pro Eye headset with tracked controllers and weapon peripheral. Middle: Images from within the VR environment showing the feed-back functionality where the user is able to watch a replay of the gaze behaviour of an expert, or themselves, from a 3^rd^ person perspective. The white lines show the gaze intersection points was traced onto the room and the red beam shows the line of sight in real-time. The user can move freely around the room to observe the replay from different angles. Bottom: Example threat and non-threat targets that appeared in the simulation. Images are from the McQueen threat assessment targets 800 series (mcqueentargets.com/products/#threat) and are reproduced with permission.

The VR environment was developed using the gaming engine Unity 2019.2.12 (Unity technologies, CA; https://unity.com/) and C#. The simulation was displayed using an HTC Vive Pro Eye headset (HTC, Taiwan; https://www.vive.com/uk/), a 6-degrees of freedom, consumer-grade VR system with a 110^o^ field of view and 90Hz refresh rate. The Vive headset had built-in binocular eye tracking capability, which sampled at 120Hz over the whole field of view to an accuracy of 0.5-1.1^o^. Gaze was calibrated in VR over 5 points prior to the room clearance tasks. The position of the headset and handheld controllers were tracked using Steam VR and the controllers were used to animate a tracked weapon in the environment (see Figure 2 top). Room scale VR was used to match the virtual room to the real space to allow participants to move freely around the environment. Graphics were generated on an HP EliteDesk PC running Windows 10, with an Intel i7 processor and Titan V graphics card (NVIDIA Corp., Santa Clara, CA) and data were recorded in csv format for offline analysis.

#### VR Room Clearance Assessment Task

The VR assessment task used for pre- and post-tests was a bespoke VR recreation of a typical synthetic room clearance training environment used in military settings and was designed with input from subject matter experts. Typical, real world synthetic environments consist of moveable walls, pieces of furniture, and static targets to allow trainees to practice a range of room configurations. The VR environment enabled a range of room configurations which reflected the different modes of room entry (depending on whether you are entering from a central door or one in the corner), and the different search procedures for different room shapes (e.g., those in an L-shape pose an additional challenge). The rooms varied in terms of numbers of targets and levels of complexity (e.g., room shape, number of targets). As a baseline, participants performed a total of 20 search iterations consisting of three different room configurations: corner fed (left and right corners), centre fed, and L-shaped (left and right L), lasting 30-60 seconds each and 10-15 minutes in total. Participants were instructed to enter the room and search the area in line with their training, then shoot any threatening targets (those pointing a weapon), while avoiding shooting non-threat targets (those not holding a weapon) (see example targets in Figure 2, bottom).

#### VR Training Conditions

Participants were randomly allocated to one of three training groups. Firstly, there was a ‘training-as-usual’ control group who completed the VR pre- and post-tests but no additional VR practice. Secondly, there was an FFEMT group who underwent two additional training sessions lasting ~20 minutes each of feed-forward instruction. Participants in this group completed further room clearances (2 x 20 repetitions) and also viewed a feed-forward animation of an idealised scan path recorded from a task expert performing the room searches (as in Figure 2, middle). The task expert was a firearms instructor with >5 years’ experience of conducting and teaching room clearance in defence and security settings. The feed-forward animation showed a live playthrough of the line of sight of the expert model and rendered gaze traces on the wall of the room. After viewing the feed-forward animation the participant would complete a room clearance of the same type. They were instructed to observe the scan path of the expert and to take note of how they searched the room. Finally, this study included an FBEMT group, who also did two further training sessions lasting around 20 minutes each. Instead of observing the expert model prior to clearing each room, however, participants in this group observed an automatic playback of their own eye movements immediately after completing each room. They were instructed to take note of how they used their eyes to search the room and to try to improve their scanning each time but were not told how to do so. The verbal instructions given to the FFEMT and FBEMT groups were as follows:

FFEMT – “*Before you enter each room you will be shown a video of an expert performing the room clearance task. This will provide an example of a good search strategy. Note how they perform a smooth sweep with their eyes to efficiently search the room and identify the targets*.’’

FBEMT – ‘‘*After you have completed each room you will be shown a replay video of your own eye movements. Take note of whether you performed a smooth efficient sweep of the room with your eyes to identify the targets*.’’

All participants continued with their normal military training regardless of their experimental group.

### Measures

#### Performance

Performance in the VR tasks (pre/post tests and training tasks) was assessed using the following dependent variables:

- Failures to inhibit fire – number of instances where shots landed on non-threatening targets, which was automatically detected by the VR simulation (calculated as a proportion of non-threat targets);
- Time to shoot all hostiles – the time from entering the room until all hostile targets had been shot, as calculated by the VR software (in seconds); and
- Missed hostiles – whether there were any threatening targets left in the room that were not successfully shot, as calculated by the VR software (as a proportion of all hostile targets).

#### Eye movement measures

*Fixation duration and search rate***.** Fixation durations and search rate have been commonly used as metrics to characterise visual search behaviour (19; 24; 57) and have been identified as markers of expertise in sporting and military activities (25; 37). Fixation duration refers to the average length of the fixations (periods in which the eye dwells in a single location) within a selected time period. Search rate is calculated from the number of fixations divided by their average duration and indicates whether the performer is using a visual strategy of a few long fixations (low search rate) or more frequent and shorter fixations (high search rate). In general, fewer fixations of longer duration are thought to indicate a more efficient and expert-like use of gaze, due to the suppression of vision during saccades (37), but this effect may be quite task-dependent and expertise has also been linked with higher search rates in some tasks (6; 57).

*Gaze Transition Entropy***.** Entropy, as defined within information theory (47), describes probabilistic uncertainty about outcomes, such that highly unpredictable outcomes or disorganised systems have high entropy. The concept of entropy has been applied to eye tracking to index the level of randomness or unpredictability in eye movements (2; 35; 40; 55). Entropy can therefore index whether a performer is performing a structured and systematic scanning pattern, or a highly variable and random one (35). To characterise the randomness of the room searches we adopted a simple measure of entropy described by Shannon and Weaver (48), known as Gaze Transition Entropy (35). Transition entropy quantifies the randomness of a scan pattern as the amount of information needed to describe it, as more random patterns require more information (measured in ‘bits’). Entropy was calculated as the sum of the probabilities of fixating each area of interest (AOI), conditional upon the previously fixated AOI:


$$Entropy\;=\sum_{i=1}^{n}p\left(i\right)\;\left[\begin{array}{c} \;\\ \begin{array}{c} \;\\ \sum_{j=1}^{n}p\;\left(\begin{array}{c} j\\ -\\ i \end{array}\right)\log_{2}p\left(\begin{array}{c} j\\ -\\ i \end{array}\right)\;\\ \;\\ \; \end{array} \end{array}\right]\;\;\;,\;i\neq j$$

where *i* represents the “from” AOI and *j* represents the “to” AOI. The AOIs in each room were defined from 12 separate segments of the room (e.g., AOI 1 was the first area on the left just inside the door and AOI 12 was the last area on the right, by the door) and the locations in which targets were present.

*Saccadic angle and intersaccadic shifts (spatial anisotropy).* Spatial anisotropy refers to whether or not saccades are ‘directionally dependent’ (3). During a task such as reading, a typical saccade profile involves saccades made left to right creating a profile that is directionally dependent (i.e., anisotropic). By contrast, a fully random search would involve saccades made in all directions. We calculated *saccadic angle* (θ) using the following formula


$$\theta^{i}=arctan\left(r_{i,y}/r_{i,x}\right)$$

where $\left(r_{i,y}/r_{i,x}\right)\;\;$are the change in the x and y components of the *i*-th saccade. Results were converted from a 180 to -180 scale so that values of θ around 180^o^ represented leftwards saccades and values near 0^o^ and 360^o^ were rightwards. Also, following Amor et al., (3) we then calculated the *intersaccadic angle*, that is the change in angle between successive saccades. From this we could identify *persistent* and *antipersistent* saccades. Persistent saccades are those that follow approximately the same direction as the preceding saccade and indicate a search that continues in a persistent direction (e.g., as in left to right reading). By contrast, antipersistent saccades ‘double-back’ on the previous saccade and indicate that the search did not continue in the same direction (3). In the present context more persistent saccades would indicate a more structured and efficient search (as per training and the scan paths of experts performing this task). Intersaccadic angle (θ_d_) was defined as


$$\theta_{d}^{i}=\arctan\left(\frac{r_{\left(i+1\right),y}}{r_{\left(i+1\right),x}}\right)-\arctan\left(\frac{r_{\left(i\right),y}}{r_{\left(i\right),x}}\right)$$

which equates to the difference between θ_d_^i+1^ and θ_d_^i^. Persistent saccades were then defined as those continuing in the same direction (within 90^o^ in either direction) and antipersistent as those that changed direction (more than 90^0^ shift).

*Time to Fixate First Target.* To assess whether participants were locating threats in the room more quickly after training, we calculated the time to fixate first target, which represented the duration (in seconds) from entering the room to when the participant’s gaze vector first intersected with a target. This was recorded automatically by the VR environment.

*Search Order Met.* An important aspect of room clearance performance is adhering to the trained search order, which starts with searching the near corner and proceeding in a systematic manner around the room. A proxy measure for correct search order was used that was based on whether participants made a fixation to the back wall before one of the corners near the door. This provided an approximate index of whether they had followed their training or if they had been distracted. This measure was calculated as the proportion of total trials in which this criteria was met (%).

### Data Analysis

Gaze data were analysed using MATLAB R2018a (Mathsworks, MA). Gaze direction data were passed through a three-frame median filter and smoothed by a second-order, zero-lag Butterworth filter with a 30Hz cut-off for fixation detection and 50Hz for saccade detection (9; 13). Next, visual fixations were identified using a spatial dispersion algorithm from the EYEMMV toolbox for MATLAB (29) by grouping successive gaze points into fixation clusters based on the their spatial similarity. Fixations were detected according to a minimum duration criterion of 100ms and spatial dispersion of 1° (as recommended in 46). A bespoke script was used to detect saccadic eye movements, in which saccades were defined as sections of data where gaze acceleration values (°/s^2^) exceeded five times the median absolute acceleration value (as in 4; 36). Saccade onset and offset times were determined from acceleration minima and maxima (13). All data as well as MATLAB analysis scripts have been made publicly available in the online repository (https://osf.io/qn2g4/).

## Results

### Pre to Post Changes in Eye Movement Metrics in VR

To determine the effect of FFEMT and FBEMT on the efficiency of visual search, a series of three (group) x two (time) ANCOVAs, with participant pool (Army/Navy) as a covariate, were run on all the eye movement measures.

### Fixation Duration

**Figure 3.**
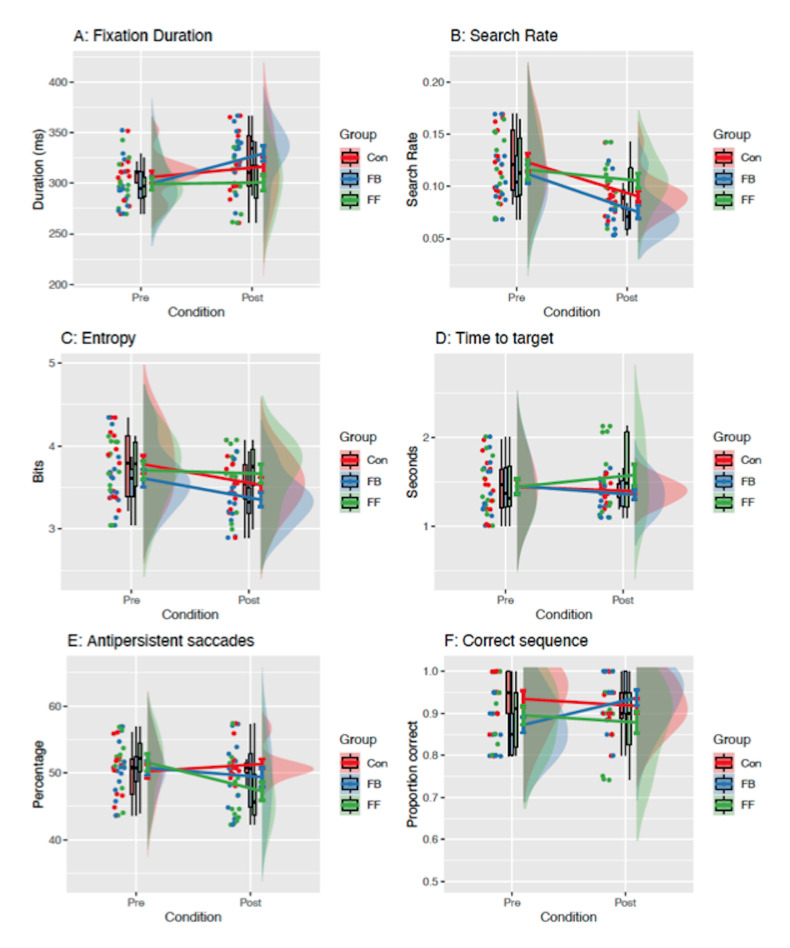
Pre- and Post-Training Eye Movement Measures in the VR Environment. Raincloud plots show the raw data points, a boxplot (black line indicates the median), and a half violin representing the distribution. Solid coloured lines show means and standard errors.

For fixation duration (see Figure 3A), the covariate was not significant [F(1,34) = 2.77, p = .11, η^2^ = 0.05] so was removed. There was found to be an overall increase in fixation durations from pre to post [F(1,35) = 11.86, p = .002, η^2^ = 0.07], but there was no difference between groups [F(2,35) = 1.40, p = .26, η^2^ = 0.05]. There was, however, a group-by-time interaction [F(2,35) = 4.27, p = .02, η^2^ = 0.05] which was explored with post-hoc tests using a Bonferroni-Holm correction for multiple comparisons. At baseline there were no differences between the groups (ps > .90). At post-test, fixation durations were significantly longer in the FBEMT than the FFEMT group (p = .045), but there were no differences observed between FBEMT and Control (p = .42) or FFEMT and Control (p = .42). Pre to post tests showed a significant increase in the FBEMT group (p = .003), but not the FFEMT (p = .85) or Control groups (p = .22), indicating that only feed-back training led to an increase in fixation durations.

### Search Rate

The covariate was again not significantly related to search rate [F(1,34) = 2.18, p = .15, η^2^ = 0.04] so was removed from the model. An overall reduction in search rate was found [F(1,35) = 35.35, p < .001, η^2^ = 0.18], but there was no effect of group [F(2,35) = 1.77, p = .19, η^2^ = 0.06]. The group-by-time interaction was very close to the significance threshold [F(2,35) = 3.19, p = .05, η^2^ = 0.03] and was therefore explored with post-hoc tests. There were no differences between groups at baseline (ps > .90). Post-training, the FBEMT group had the lowest search rate, which was significantly lower than FFEMT (p = .01), but not significantly different from Control (p = .16). There was no significant difference between FFEMT and Control (p = .16). Comparisons of pre to post changes showed significant reductions for Control and FBEMT (ps = .003) but not for FFEMT (p = .27). This result aligns with the increase in mean fixation duration for FBEMT but not FFEMT (see Figure 3B).

### Gaze Transition Entropy

For entropy (see Figure 3C), the covariate was not significant [F(1,34) = 0.13, p = .74, η^2^ = 0.00] so was removed. There was an overall reduction in entropy from pre to post [F(1,35) = 9.33, p = .004, η^2^ = 0.06], but there was no effect of group [F(2,35) = 1.60, p = .22, η^2^ = 0.06], and no group-by-time interaction [F(2,35) = 1.35, p = .27, η^2^ = 0.02]. This suggests that all groups learned more structured search patterns over time and that the addition of feed-forward or feed-back eye movement training did not significantly accelerate this learning.

### Time to Fixate First Target

The covariate participant pool was not significant [F(1,34) = 4.93, p = .52, η^2^ = 0.03] so was removed. There was no overall change in the time to fixate the first target from pre to post [F(1,35) = 0.01, p = .93, η^2^ = 0.00], no effect of group [F(2,35) = 0.89, p = .42, η^2^ = 0.03] and no group-by-time interaction [F(2,35) = 1.15, p = .33, η^2^ = 0.03] (see Figure 3D). 

### Percentage of Antipersistent Saccades

The participant pool covariate was significant for antipersistent saccades [F(1,34) = 4.33, p = .045, η^2^ = 0.07] so was retained in the ANCOVA model. There was no overall change in the percentage of antipersistent saccades from pre to post [F(1,34) = 2.30, p = .14, η^2^ = 0.02], and no effect of group [F(2,34) = 0.55, p = .59, η^2^ = 0.02]. There was, however, a narrowly significant group-by-time interaction [F(2,34) = 3.54, p = .04, η^2^ = 0.07]. Post-hoc tests with Bonferroni-Holm correction indicated no differences between groups at baseline (ps = 1.00). Post training, there was a significant difference between FFEMT and Control (p = .03), but not between FFEMT and FBEMT (p = .48) or FBEMT and Control (p = .48) groups. This result indicates that participants given FFEMT subsequently made the least return saccades to search already viewed areas of the room (see Figure 3E).

### Search Order Compliance

The covariate participant pool was significant [F(1,34) = 0.42, p = .52, η^2^ = 0.03] so was retained in the ANCOVA model. There was no overall change in the search order measure from pre to post [F(1,34) = 0.01, p = .92, η^2^ = 0.00], and no effect of group [F(2,34) = 1.88, p = .17, η^2^ = 0.05] (see Figure 3F). The group-by-time interaction was close to the significance threshold [F(2,34) = 3.00, p = .06, η^2^ = 0.07] so post-hoc tests were run. The pairwise comparisons showed no differences at either baseline (ps > .06) or post-training (ps = .21), but the interaction was driven by a significant increase in how often the search order was met in the FBEMT group (p = .05) but not the FFEMT (p = .93) or Control (p = .93) groups.

### Changes in Performance Within the VR Environment

Some of the performance variables displayed deviations from normality but parametric tests were still used, as Analysis of Variance (ANOVA) is largely robust to such deviations (44).

**Figure 4.**
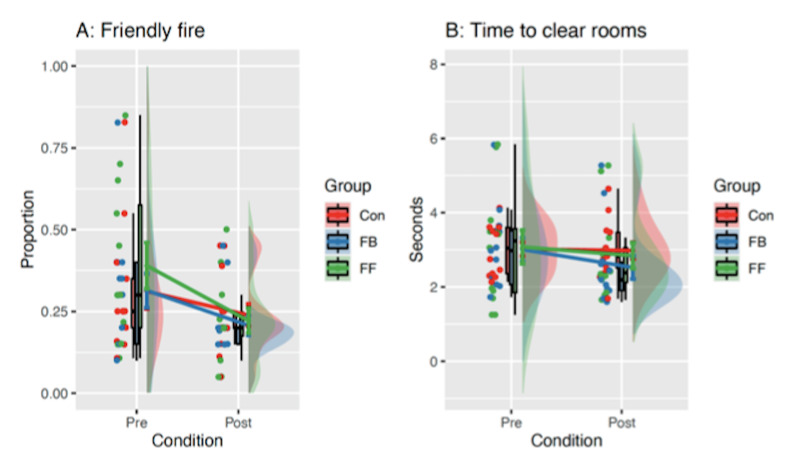
Pre- and Post-Training Performance Measures in the VR Environment. Raincloud plots show the raw data points, a boxplot (black line indicates the median), and a half violin representing the distribution. Solid coloured lines show means and standard errors.

To assess whether there was an effect of the different training groups on room clearance performance in the VR environment, a series of three (group) x two (time) Analysis of Covariance (ANCOVA) models were run on performance measures (see Figure 4). Participant pool (Urban Instructors / Royal Marines) was initially entered as a covariate to account for any differences between the participant groups, but only retained in the model if it showed a significant relationship with the dependent variable.

### Failures to Inhibit Fire

The participant pool covariate did not have a significant relationship with failure to inhibit fire [*F*(1,34) = 0.06, *p* = .81, *η*^2^ = 0.00] so was removed from the model. The ANOVA showed an overall reduction in failures to inhibit fire [*F*(1,35) = 15.47, *p* < .001, *η*^2^ = 0.11], but there was no effect of group [*F*(2,35) = 0.31, *p* = .73, *η*^2^ = 0.01], and no group-by-time interaction [*F*(2,35) = 0.89, *p* = .42, *η*^2^ = 0.01], suggesting that this aspect of performance improved similarly across all training groups.

### Time to Shoot All Hostiles

For the variable time to shoot hostiles, the covariate participant pool was significant [*F*(1,34) = 4.69, *p* = .04, *η*^2^ = 0.09] so was retained. There was no overall change in time to clear hostiles from pre to post [*F*(1,34) = 0.49, *p* = .49, *η*^2^ = 0.00], no effect of group [*F*(2,34) = 0.37, *p* = .69, *η*^2^ = 0.02], and no group-by-time interaction [*F*(2,34) = 0.52, *p* = .60, *η*^2^ = 0.01].

### Proportion of Hostiles Cleared

The participant pool covariate was not significant [*F*(1,34) = 1.58, *p* = .22, *η*^2^ = 0.00] so was removed. There was an overall increase in the percent of hostiles cleared [*F*(1,35) = 9.22, *p* = .005, *η*^2^ = 0.11], but there was no effect of group [*F*(2,35) = 0.10, *p* = .91, *η*^2^ = 0.00], and no group-by-time interaction [*F*(2,35) = 0.50, *p* = .61, *η*^2^ = 0.01], suggesting that all participants improved similarly and there was little additive effect of eye movement training for this measure.

**Table 2.** Summary of the dependent variables and the significant pairwise comparisons

	Fixation duration		Search rate		Antipersistent saccades		Search order compliance	
	Pre	Post	Pre	Post	Pre	Post	Pre	Post
*FFEMT v FBEMT*	.89	**.045**	1.00	**.01**	1.00	.48	.41	.072
*FFEMT v Control*	.89	.42	1.00	.16	1.00	**.03**	.32	.213
*FBEMT v Control*	.89	.42	1.00	.16	1.00	.48	.07	.453
	Pre-post change		Pre-post change		Pre-post change		Pre-post change	
*FFEMT*	.85		.27		**.02**		.93	
*FBEMT*	**.003**		**.003**		.35		**.05**	
*Control*	.22		**.003**		.40		.93	

## Discussion

In the present work we sought to explore the use of eye movement training as a method for accelerating the learning of visuomotor and decision-making skills in a military context. Most eye movement training research has focused on feed-forward eye movement training methods (14; 17; 26; 56), so we aimed to extend the literature by also assessing the potential of *feed-back* training where the participant learns from replays of their own eye movements. As immersive technologies are providing many opportunities for human skills training, we also aimed to understand whether these methods could be effective when integrated into VR. As predicted, there were changes in eye movements that suggested more efficient visual search behaviours as a result of the eye movement training, but there was no indication that this was accompanied by measurable changes in performance outcomes.

Comparisons of gaze fixation durations during the room clearance task showed an overall increase in mean durations and a statistical interaction effect indicated that the greatest increases were observed in the FBEMT group. Likewise, there was an overall reduction in search rate with the largest changes in the FBEMT group. This suggests that observing one’s own eye movements may have generated a visual control strategy characterised by fewer fixations of longer duration. These are markers that have previously been associated with perceptual-cognitive expertise and a more efficient use of vision (25; 37).

Participants’ visual scan paths also improved over the course of training, although these changes inconsistently varied between groups. For gaze entropy, values generally decreased from pre- to post-training, which suggests that scanning became less variable and more structured over time. However, no significant differences emerged between training groups for this metric. Conversely, for the antipersistent saccades measure, which characterised how often a saccade ‘doubled-back’ on the previous gaze shift, an interaction effect indicated that there may have been a reduction in antipersistent (i.e., return) saccades in the FFEMT group *only*. This finding is consistent with our expectation that trainees in this group would adopt the search characteristics of the expert model, who made very few gaze shifts back towards previously searched areas. There was no change in the time taken to fixate the first target. There was also no overall improvement in how often the search order criteria was met, but there was an increase in this measure for the FBEMT group. In summary, there were beneficial effects of both the eye movement training approaches, which appear to have prompted slightly different changes in gaze control throughout the short period of training.

The varied gaze adaptations may be due to different learning mechanisms underpinning FFEMT and FBEMT. The effects of FBEMT may be similar to the development of error detection and correction mechanisms that have been cited as responsible for the effectiveness of observational learning from watching one’s own mistakes (7; 20). The adoption of expert-like behaviours during FFEMT are, however, likely to reflect the acquisition of more efficient visual guidance through more implicit means (52). It has been suggested that FFEMT works much like ‘cueing’, which aims to orient the trainees attention to the most important areas of the visual scene (12; 26). However, this assumes that the expert model is always looking at the most relevant information, which may not be the case. Although the current research does not allow for further elucidation of the different mechanisms involved in FBEMT and FFEMT, future research should seek to explore this, and investigate whether they can have complimentary effects when combined in practice.

For performance measures, statistical tests showed that there were general reductions in failures to inhibit fire (i.e., fewer non-threat targets were shot) and increases in the proportion of hostiles cleared (i.e., fewer threats were missed), but that the size of the improvements did not differ between training groups. This indicates that all participants improved in these aspects regardless of the training that they were assigned. One reason for the lack of performance effects may be the relatively short training durations. We only used two short sessions here (~20 minutes each) which was sufficient for changes in eye movements but may not have been a sufficient ‘dose’ for performance differences to emerge. In previous eye movement training literature, the effect size of eye movement changes can be 2-3 times the size of performance changes (41; 52), so may be more easily detected. Consequently, full training effects may not have been captured in this study and future research should aim to adopt longer durations of training, where larger training effects may emerge. The lack of performance effects could also be due to a lack of sensitivity in the performance measures. While many eye movement training studies have used constrained visuomotor skills (52) or decision making tasks (14), the room search task used here was a complex combination of the two. Consequently, it may be difficult to detect changes in these global decision processes from relatively subtle changes in eye movements over such a short period of time.

There is another important limitation to consider when interpreting the current findings. The relevant causal comparator for this work with an applied focus was training as usual (28), so our conclusions are limited by the nature of the control group. Any effects of the FFEMT and FBEMT training could be related to simply practicing the VR room clearance task. As the aim of this work was to begin exploring the potential of FFEMT and FBEMT as an *addition* to current training, rather than a replacement, the training as usual control group was appropriate, but future work should consider alternative control conditions to examine the mechanisms of effect more closely.

In terms of practical applications, this work has suggested that there are opportunities for using VR to monitor performance and provide automated feedback in applied training settings like the military. This type of approach can reduce the need for specialist trainers to be present and allow effective practice with reduced input of resources. It is worth noting however, that the presence of an expert trainer to provide additional feedback could well have accelerated the learning in VR. Future research may wish to examine the degree to which users benefit from external feedback in this way, to identify whether VR and eye movement training methods require expert assistance (or can be performed in isolation).

### Conclusions

There is a large body of previous work that supports the effectiveness of eye movement training in visuomotor and decision-making skills – e.g., in sport, surgery, and previous military tasks (42; 53). Here we extended this literature by demonstrating the potential of feed-back eye movement training in a military context, as well as integrating both feed-forward and feed-back principles within VR. Even though no obvious performance improvements were observed, the adaptive changes in gaze efficiency that were obtained from two short training sessions suggests that there is encouraging potential for using both FBEMT and FFEMT within VR

### Ethics and Conflict of Interest

The author(s) declare(s) that the contents of the article are in agreement with the ethics described in http://biblio.unibe.ch/portale/elibrary/BOP/jemr/ethics.html and that there is no conflict of interest regarding the publication of this paper.

### Acknowledgements

This work was funded by the Defence Science and Technology Laboratory via the Human Social Science Research Capability framework (HS1.010).

### Data

All relevant data and code is available online from: https://osf.io/qn2g4/
